# Sustained Reductions in Online Search Interest for Communicable Eye and Other Conditions During the COVID-19 Pandemic: Infodemiology Study

**DOI:** 10.2196/31732

**Published:** 2022-03-16

**Authors:** Michael S Deiner, Gerami D Seitzman, Gurbani Kaur, Stephen D McLeod, James Chodosh, Thomas M Lietman, Travis C Porco

**Affiliations:** 1 Francis I Proctor Foundation University of California San Francisco San Francisco, CA United States; 2 Department of Ophthalmology University of California San Francisco San Francisco, CA United States; 3 School of Medicine University of California San Francisco San Francisco, CA United States; 4 Department of Ophthalmology Massachusetts Eye and Ear Harvard Medical School Boston, MA United States; 5 Department of Epidemiology and Biostatistics Global Health Sciences University of California San Francisco San Francisco, CA United States

**Keywords:** COVID-19, pandemic, communicable disease, social distancing, infodemiology, Google Trends, influenza, conjunctivitis, ocular symptoms, seasonality, trend, online health information, information-seeking

## Abstract

**Background:**

In a prior study at the start of the pandemic, we reported reduced numbers of Google searches for the term “conjunctivitis” in the United States in March and April 2020 compared with prior years. As one explanation, we conjectured that reduced information-seeking may have resulted from social distancing reducing contagious conjunctivitis cases. Here, after 1 year of continued implementation of social distancing, we asked if there have been persistent reductions in searches for “conjunctivitis,” and similarly for other communicable disease terms, compared to control terms.

**Objective:**

The aim of this study was to determine if reduction in searches in the United States for terms related to conjunctivitis and other common communicable diseases occurred in the spring-winter season of the COVID-19 pandemic, and to compare this outcome to searches for terms representing noncommunicable conditions, COVID-19, and to seasonality.

**Methods:**

Weekly relative search frequency volume data from Google Trends for 68 search terms in English for the United States were obtained for the weeks of March 2011 through February 2021. Terms were classified a priori as 16 terms related to COVID-19, 29 terms representing communicable conditions, and 23 terms representing control noncommunicable conditions. To reduce bias, all analyses were performed while masked to term names, classifications, and locations. To test for the significance of changes during the pandemic, we detrended and compared postpandemic values to those expected based on prepandemic trends, per season, computing one- and two-sided *P* values. We then compared these *P* values between term groups using Wilcoxon rank-sum and Fisher exact tests to assess if non-COVID-19 terms representing communicable diseases were more likely to show significant reductions in searches in 2020-2021 than terms not representing such diseases. We also assessed any relationship between a term’s seasonality and a reduced search trend for the term in 2020-2021 seasons. *P* values were subjected to false discovery rate correction prior to reporting. Data were then unmasked.

**Results:**

Terms representing conjunctivitis and other communicable conditions showed a sustained reduced search trend in the first 4 seasons of the 2020-2021 COVID-19 pandemic compared to prior years. In comparison, the search for noncommunicable condition terms was significantly less reduced (Wilcoxon and Fisher exact tests, *P*<.001; summer, autumn, winter). A significant correlation was also found between reduced search for a term in 2020-2021 and seasonality of that term (Theil-Sen, *P*<.001; summer, autumn, winter). Searches for COVID-19–related conditions were significantly elevated compared to those in prior years, and searches for influenza-related terms were significantly lower than those for prior years in winter 2020-2021 (*P*<.001).

**Conclusions:**

We demonstrate the low-cost and unbiased use of online search data to study how a wide range of conditions may be affected by large-scale interventions or events such as social distancing during the COVID-19 pandemic. Our findings support emerging clinical evidence implicating social distancing and the COVID-19 pandemic in the reduction of communicable disease and on ocular conditions.

## Introduction

Infodemiology, an emerging field of study within health informatics, applies the science of distribution and determinants of information in an electronic medium such as the internet or within a population toward informing public health and policy [1-4]. The COVID-19 pandemic highlights the utility of infodemiology from the ability to predict outbreaks of coronavirus infection based on internet search engine queries, social media post–based syndrome surveillance, and search engine data mining to cluster query and click data as an estimate of the prevalence of symptoms patients sought to address outside of clinical appointments or business hours [1,2,5-7]. Although no standard methodologic approach has been established in the past decade, recently new standardized infodemiologic study methods have been proposed to strengthen the validity and utility of its application in health [8].

Google Trends has emerged as a predictive tool for disease occurrence and outbreaks. For example, one study demonstrated a strong correlation between keyword-triggered link click counts on Google and influenza cases 1 week later as the 2004-2005 Canadian influenza season unfolded (Pearson correlation coefficient *r*=0.91) [9]. Infodemiologic approaches such as use of Google Trends unlocks access to real-time predictive analysis of health-related behaviors. This was previously unfathomable, when much of public health analytics was predicated on collecting and sifting through large data sets [2,10]. For example, social media–based surveillance of foodborne diseases have been shown to be 66% effective, more rapid, and cheaper than these data-based surveillance methods [11].

Clinical studies of the COVID-19 pandemic have suggested potential links between the pandemic with changes in health conditions [12-21]. This includes studies and reports on ocular symptoms and health [22-36]. Online searches and social media reflect the clinical seasonality and epidemics of conjunctivitis [37-40]. Previously, we found evidence that during the start of the COVID-19 pandemic (through April 2020), some ocular-related terms (in multiple languages on a worldwide level) showed an increased search trend. These terms included “burning,” “sore,” and “red” eyes [5]. Subsequently, other studies of search data through June 2020 found a strong correlation between some ocular search terms and cases of COVID-19 on a country level in Europe [41]. In our prior study, searches for English-language conjunctivitis- and pink eye–related terms in March and April 2020 were lower compared with those in prior years. We had conjectured that one cause of these search trend results could be that implementation of school closures and social distancing starting in March 2020 had reduced the incidence of contagious conjunctivitis cases, resulting in reduced information-seeking about conjunctivitis [5]. However, our findings were limited as our study data time series ended quite early into the pandemic in April 2020.

In this study, using masked analyses of searches geolocated to the United States for 1 full year after the pandemic began, we assessed whether a reduction in searching occurred for conjunctivitis in the United States compared to the prior 9 years. We then assessed whether this was sustained for multiple seasons throughout the COVID-19 pandemic in 2020-2021. We also assessed whether the search volume decreased for other common school- and workplace-based communicable diseases, including strep throat, chicken pox, the common cold, as well as other conditions of acute exposure such as sexually transmitted diseases (STDs) and bug bites. We compared the results for that class of terms (referred to as “communicable”) to searches for control “noncommunicable” conditions, including some ocular terms for which we and others had previously found had increased search activity at the start of the pandemic [5,41]. We also assessed whether terms with stronger seasonal variation were more likely to have a decreased search trend during the COVID-19 pandemic. In addition, we assessed whether there was sustained change across multiple seasons, compared to the prior 9 years, for the group of terms we had classified as COVID-19 pandemic–related (related to a search about distinguishing or identifying COVID-19 symptoms).

## Methods

### Google Search Data

Weekly relative search frequency volume data for search terms in English for the United States were obtained on March 9, 2021, for the weeks of March 1, 2011, through February 28, 2021, as previously described using the Google Health application programming interface (API) [40,42-44]. This provided a long baseline of prepandemic data as a basis for comparisons (described below). Queries of this API allow specification of the following: a set of search terms (eg, “*coronavirus symptoms,*” “*shingles treatment*”), time range (start date and end date), interval (*day, week, month*), and geolocation (eg, “*United States*”). For any given query, for each search term, a search activity value is provided at each time interval, which represents the relative share of search for that term in proportion to all Google searches that were made within the specified time range and geolocation [45]. Search terms were chosen based on our prior studies [5], COVID-19, and on common terms used in the United States for communicable and noncommunicable conditions. Some terms served as a surrogate for ambiguously named conditions to improve the health-specificity of search data (eg, we used “*cold medicine*” for the common cold and “*shingles treatment*” for shingles). Classifications were assigned a priori. We classified 16 terms as COVID-19 pandemic–related conditions, including respiratory, allergic, or flu-like terms (as we assumed they may represent a symptomatic search for those affected by, or initially concerned about, COVID-19). We also included 29 terms that were classified as communicable (communicable conditions unrelated to the COVID-19 pandemic) and 23 terms that were classified as noncommunicable (control noncommunicable conditions, less likely related to the COVID-19 pandemic).

### Masking of Terms, Classifications, and Location

To reduce bias, actual search terms were masked using numeric codes before the data were analyzed. To further mask, data for the same terms for two other masked countries were also included and names of our assigned classification groups were also encoded. In this way, individuals assessing statistical outcomes were naïve to the actual terms and to their assigned classifications, as well as to the country of search term origin.

### Statistical Analysis

#### Overview

The masked statistical analysis, described in detail below, included identifying seasonal search features for each term. It also included fitting models for spring (March to May 2020), summer (June to August 2020), autumn (September to November 2020), and winter (December to February 2020-2021). This was done to contrast search interest during each season of the first year of the pandemic with that of the same season from the prior 9 years, by identifying seasons for each term that differed (as well as those that were specifically reduced) during the pandemic compared to the prior 9 years. We then compared those results for terms representing different classes of conditions, as well as to the seasonality of terms, as described in detail below.

#### Analysis of Changes in Search Trends in 2020-2021 Seasons Compared to Prior Years

To test for the significance of changes in the period following March 2020, the following algorithm was used. Time series were first subject to the Hampel filter for outlier removal (R package *pracma*). For more complete series (time series with fewer than 20% missing data), we detrended the time series using the residuals from Theil-Sen regression with respect to the calendar time for the pre-COVID-19 epoch (March 2011 to February 2020). The 9 years of pre-COVID-19 time-series data were intended to provide sufficiently precise estimations of prepandemic seasonal and secular trends for our planned comparison of these features during the pandemic period. Theil-Sen regression is a nonparametric fixed-effects regression model designed to minimize the influence of outliers [46,47]. Thus, when sufficient data were available, we compared postpandemic values to what would have been expected based on prepandemic trends, as has been done in other studies (eg, [48,49]). We then compared the levels of search for spring 2020 (and the other seasons) to the pre-COVID-19 trend line as follows. We applied a robust linear mixed-effects regression model to compare the residuals of observations for each season, thus comparing the levels for spring 2020, summer 2020, autumn 2020, and winter 2021 to the corresponding times of previous years. Using this model, we computed both one- and two-sided *P* values. Significant two-sided *P* values represented a *P* value for a search change (increase or decrease) in 2020-2021 compared to prior years. Significant one-sided *P* values represented a *P* value for search reduction in 2020-2021 compared to prior years. For time series containing more than 20% missing (or zero) data, we performed robust mixed-effects regression using indicators for spring, summer, autumn, and winter of 2020 as predictors (clustering on year); one- and two-sided *P* values were computed using the standard normal distribution. This analysis only compared values for each season after the pandemic began to those before. We interpreted all significant two-sided *P* values as indicating an increase if significance was not also seen using the one-sided tests specific to identifying decreases. All computations were performed using R for MacIntosh v.4.0 (R Foundation for Statistical Computing, Vienna, Austria); the R packages *pracma*, *mblm,* and *robustlmm* were used for Hampel filter, Theil-Sen, and robust linear mixed models, respectively [50-52].

#### Comparing Changes in Searches in 2020-2021 Seasons for Communicable Versus Noncommunicable and Non-COVID-19 Classification Groups

We then performed an analysis of the previously calculated *P* values for search reduction by term groups to ask if non-COVID-19 terms representing communicable disease were more likely to show significant reductions in searches in 2020-2021 than noncommunicable terms. We compared the *P* values for search reduction between these two groups using the Wilcoxon rank-sum test. Similarly, we assessed the binary classification of significance at the .05 level using the Fisher exact test (where a significant *P* value indicates a difference in the proportion of *P* values less than .05 found between the two groups).

#### Determining Seasonal Characteristics and Their Relationships to Search Reductions in 2020-2021

Standard circular statistical methods were used for seasonal analysis, computing the circular mean, a measure of central tendency for the occurrence time of searches within the yearly cycle [53]. We also report the amplitude-to-mean (AtM) ratio (ie, the ratio of the difference between the peak and the mean to the mean itself) as an estimate of the degree of seasonality. Large AtM values correspond to large swings or oscillations, while small values correspond to minor fluctuations on a yearly cycle. Statistical significance of seasonality per term was assessed using Morlet wavelets, reporting the largest daily *P* value for the power at the annual cycle over the course of the time series (excluding the first and last years) [44,54]. This provided a conservative requirement for consistency of the annual cycle for all years. Calculations were performed using the R package *WaveletComp* [55]. Using the *P* values reflecting seasonality for a term, for each season, we then also assessed if there was a relationship between the *P* value for search reduction in 2020-2021 and the seasonal *P* value for that term. This was assessed using Theil-Sen regression.

#### Unmasking, Describing, and Visualizing Results

After all statistical analyses were completed, search terms, country, and classifications were then unencoded (unmasked). The weekly (x axis) and resulting mean search interest values (y axis) for terms were plotted. Weekly data were plotted as log-transformed Hampel-smoothed raw mean values+1 for improved scaling and visualizations. Seasons are indicated with vertical dashed line separators. The 2020 weekly mean search values are plotted as a red solid line, 2021 values are plotted as a red dashed line, 2017-2019 plots are gold, 2014-2016 data are green, and 2011-2013 data are blue. *P* values at the top of each panel for any season indicate if searches in 2020-2021 were significantly different overall (red, *P* values for search change) or specifically lower (blue, *P* values for search reduction), compared to those in the same quarters in 2011-2019 (differences significant at *P*>.05 are presented in tables). In addition, the overall seasonality is presented for each term (black text on the lower left of each panel in figures), indicating if a term is significantly seasonal. If significantly seasonal (defined as *P*<.05), the AtM (as an indicator of relative seasonal strength) and a circular mean week (as an indicator of the peak high season) are provided. All of the statistical values described above are included in figures and all *P* values are also presented in tables. We subjected *P* values to false discovery rate correction prior to reporting.

### Ethics Considerations

This study received approval from the University of California San Francisco institutional review board (14-14743) and adhered to the Declaration of Helsinki.

## Results

### Overview of Changes in Search Trends in 2020-2021 Seasons Compared to Prior Years

Overall, we found that at the start of the pandemic (spring 2020), many terms of all three classifications appeared to have search patterns that differed from those in prior years. Some changes persisted for subsequent seasons. Further details and statistical analysis results are described below first for COVID-19–related terms and then for non-COVID-19–related terms (including comparison of search term groups classified as representing communicable conditions vs noncommunicable conditions).

### COVID-19–Related Search During the Pandemic

**Figure 1:**
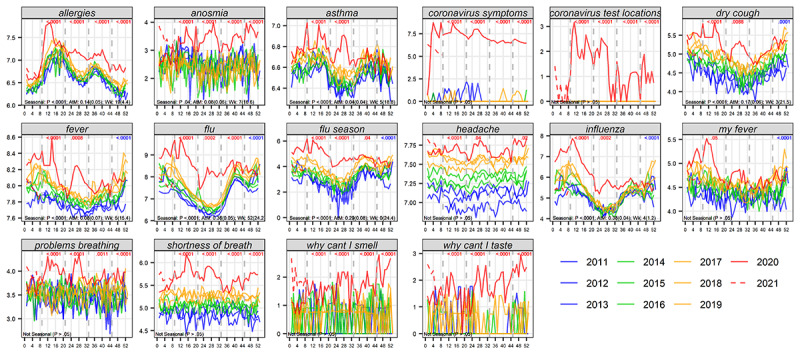
Search interest for COVID-19–related terms in 2020-2021 seasons compared to the same seasons in 2011-2019. In each panel, the x axis indicates week of the year and the y axis indicates weekly mean search interest values (Hampel-filtered and log-transformed for presentation purposes) for that term. Solid red, 2020 values; dashed red, 2021 values; gold, 2017-2019; green, 2014-2016; blue, 2011-2013. The 4 seasons are separated with vertical dashed lines. *P* values at the top of each panel for each season indicate if searches in that season of 2020-2021 were significantly (P<0.05) different overall (red, 2-sided test) than the same quarters in 2011-2019. Significant reductions are indicated by blue *P* values. Nonsignificant (*P*>.05) values are not shown. Seasonal characteristics for each term are shown as black text on the lower left of each panel. For terms with seasonality (*P*<.05), amplitude to mean ratios (AtM) are provided as an indicator of relative seasonal strength, as are circular mean week (Wk.) as an indicator of peak high season (assuming annual seasons); standard deviations are in parentheses.

Of the terms we had a priori classified as COVID-19 pandemic–related, resulting quarterly *P* values for the search change and for search reduction, as well as plotted data, indicated significant search increases compared to prior years. Of note, this group of terms includes those we classified as *potentially related*, due to the public’s concern about conditions with symptoms similar to those of COVID-19 (such as flu and allergy). Increases were observed for spring and summer 2020-2021 and often in additional seasons. A common exception was that several potentially flu-related terms switched to a significant decrease in winter 2020 (*P*<.001) (see Figure 1 and Table 1).

**Table 1:** Search interest for COVID-19–related terms in 2020-2021 seasons compared to the same seasons in 2011-2019 (related to Figure 1).

Terms	Seasonality^a^				*P* value^b^ for test of different search from prior years for each term					*P* value^c^ for test of lower search from prior years for each term			
	*P* value	AtM^d^, mean (SD)	Week^e^, circular mean (SD)	Spring		Summer	Autumn	Winter	Spring		Summer	Autumn	Winter
allergies	<.001	0.14 (0.05)	19 (4.4)	<.001		<.001	<.001	<.001	>.99		>.99	>.99	>.99
anosmia	.04	0.08 (0.06)	7 (16.6)	<.001		<.001	<.001	<.001	>.99		>.99	>.99	>.99
asthma	<.001	0.04 (0.04)	5 (18.8)	<.001		<.001	.13	.25	>.99		>.99	.11	.17
coronavirus symptoms	>.99	—^f^	—	<.001		<.001	<.001	<.001	>.99		>.99	>.99	>.99
coronavirus test locations	>.99	—	—	<.001		<.001	<.001	<.001	>.99		>.99	>.99	>.99
dry cough	<.001	0.17 (0.06)	3 (21.5)	<.001		.009	.33	<.001	>.99		>.99	.27	<.001
fever	<.001	0.14 (0.05)	19 (4.4)	<.001		<.001	<.001	<.001	>.99		>.99	>.99	>.99
flu	<.001	0.36 (0.05)	52 (24.2)	<.001		<.001	<.001	<.001	>.99		>.99	>.99	<.001
flu season	<.001	0.29 (0.08)	0 (24.4)	<.001		<.001	.04	<.001	>.99		>.99	>.99	>.99
headache	.22	—	—	<.001		.04	.43	.02	>.99		>.99	>.99	>.99
influenza	<.001	0.38 (0.04)	4 (1.2)	<.001		<.001	.10	<.001	>.99		>.99	>.99	<.001
my fever	.13	—	—	.05		.13	.86	<.001	>.99		>.99	.66	<.001
problems breathing	>.99	—	—	<.001		<.001	<.001	<.001	>.99		>.99	>.99	>.99
shortness of breath	.43	—	—	<.001		<.001	<.001	<.001	>.99		>.99	>.99	>.99
why cant I smell	>.99	—	—	<.001		<.001	<.001	<.001	>.99		>.99	>.99	>.99
why cant I taste	.61	—	—	<.001		<.001	<.001	<.001	>.99		>.99	>.99	>.99

^a^Indicates if the search for the years 2011-2019 shows a significant (*P*<.05) seasonal trend.

^b^Two-sided *P* values regarding any change in search from prior years for each season.

^c^One-sided *P* values regarding a decrease in search from prior years for each season.

^d^AtM: amplitude to mean ratio, indicating relative seasonal strength.

^e^Indicates peak high season.

^f^Not applicable; AtM and circular mean values are provided only for search terms where statistical evidence of that term being seasonal was found.

### Changes in Searches in 2020-2021 Seasons for Communicable Versus Noncommunicable and Non-COVID-19 Classification Groups

The two ocular terms we had classified a priori as communicable, “conjunctivitis” and “pink eye,” both had significant reductions for all 4 seasons of 2020-2021 (*P*<.001) compared to prior years. Overall, in 2020-2021, these and other communicable condition search terms appeared to have more reductions in search compared with the reductions in control noncommunicable terms. To test this hypothesis further, we compared the *P* values for search changes and reductions between the communicable and noncommunicable class of terms (excluding COVID-19–related terms).

**Figure 2:**
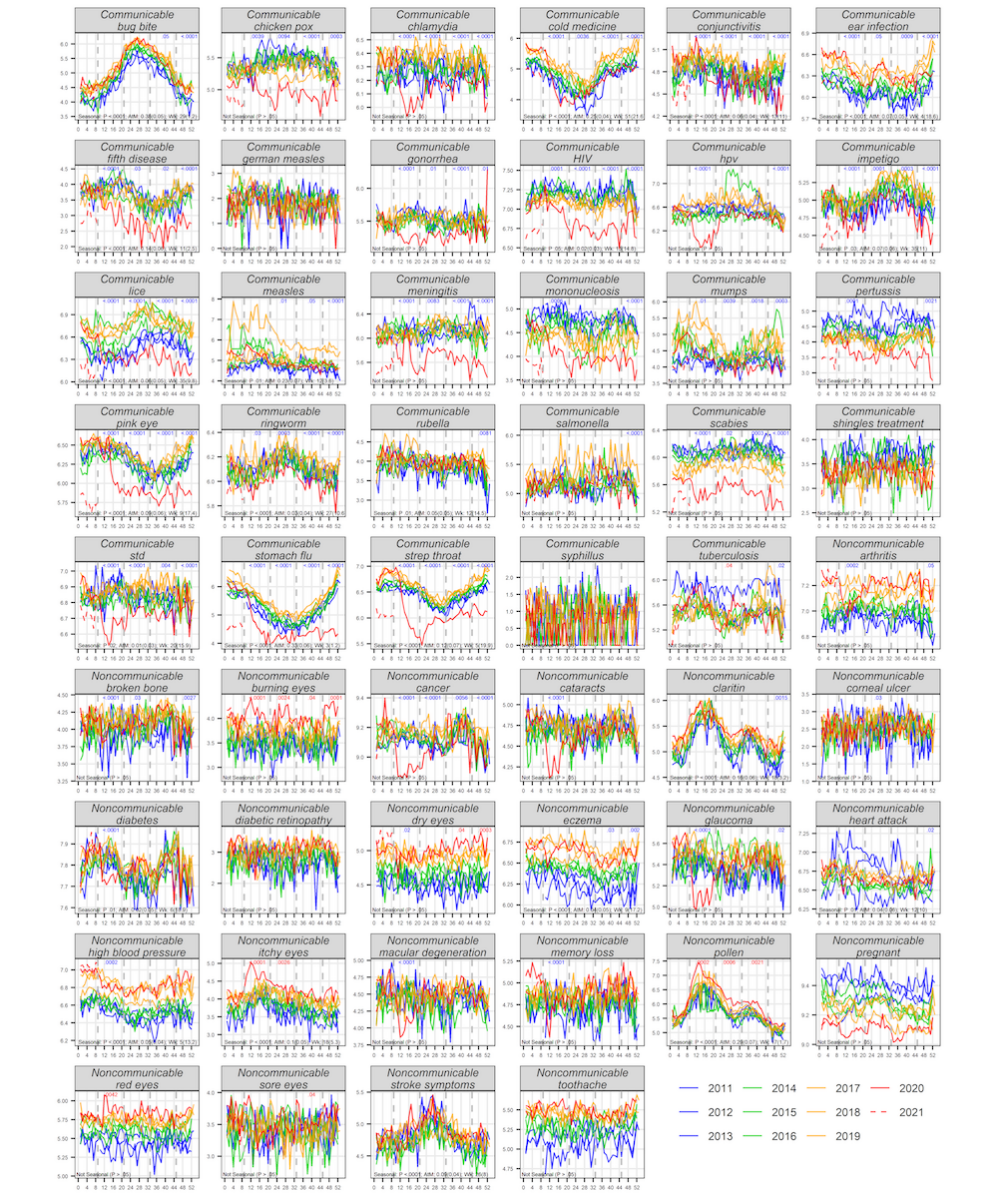
Search interest for non-COVID-19, communicable, and noncommunicable terms in 2020-2021 seasons compared to the same seasons in 2011-2019. Time-series annual mean weekly search interest; *P* values indicating changes in 2020-2021 and seasonal values are all as described for Figure 1. Panel labels indicate communicable (shown first) and noncommunicable (shown second) classes that were compared group-wise using the Wilcoxon rank-sum test and Fisher exact test (described in the text and in Tables 2 and 3).

We first assessed if *P* values for the search change in the non-COVID-19 communicable term group differed significantly from *P* values for the search change in the noncommunicable group (Figure 2, red *P* values; Table 2 "different search from prior years"). In spring 2020, we found no evidence for a significant difference between these groups for the *P* values (Wilcoxon rank-sum test, *P*=.99) or in the proportion of search terms with significant *P* values (Fisher exact test, *P*=.83). In contrast, for the subsequent 3 seasons in 2020-2021, the levels of searches were significantly different in 2020-2021 (compared to past years) for the communicable versus control noncommunicable groups of terms. This was observed when comparing the *P* values per group (Wilcoxon rank-sum test: summer *P*=.05, autumn *P*=.02, winter *P*=.006). Similarly, the proportion of search terms with significant search changes in 2020-2021 was significantly higher for the communicable group compared with the noncommunicable group (Fisher exact test: summer *P*=.01, autumn *P*=.01, winter *P*=.003).

We also assessed specifically if significant reductions in search differed for the communicable and noncommunicable classifications of non-COVID-19 term groups. To do so, we compared the *P* values for search reduction (see Table 2, “lower search from prior years”) between groups, by season. We found little evidence for a significant difference in overall reductions in search between these groups in spring 2020 (Wilcoxon rank-sum test: *P*=.04, Fisher exact test: *P*=.09). For each of the subsequent 3 seasons in 2020-2021, the levels of search were much more significantly reduced in 2020-2021 (compared to past years) for the communicable class of terms than for the noncommunicable term group (Wilcoxon rank-sum test: summer *P*<.001, autumn *P*<.001, winter *P*<.001; Fisher exact test: summer *P*<.001, autumn *P*<.001, winter *P*<.001).

The Wilcoxon rank-sum test and Fisher exact test *P* values for the overall differences in search postpandemic between the two classification groups (communicable and noncommunicable conditions) per season, described above, are shown in Table 3.

**Table 2:** Search interest for non-COVID-19 communicable versus noncommunicable term groups in 2020-2021 seasons compared to those seasons in 2011-2019.

Term		Seasonality^a^				*P* values^b^ for test of different search from prior years for each term					*P* values^c^ for test of lower search from prior years for each term			
		*P* value	AtM^d^, mean (SD)	Week^e^, circular mean (SD)	Spring		Summer	Autumn	Winter	Spring		Summer	Autumn	Winter
**Communicable and/or acute exposure conditions (non-COVID)**														
	bug bite	<.001	0.38 (0.05)	29 (1.2)	.54		.43	.06	<.001	.41		>.99	.05	<.001
	chicken pox	.06	—^f^	—	<.001		<.001	<.001	<.001	<.001		.009	<.001	<.001
	chlamydia	.83	—	—	<.001		<.001	<.001	<.001	<.001		<.001	<.001	<.001
	cold medicine	<.001	0.25 (0.04)	51 (21.6)	<.001		.003	<.001	<.001	<.001		.004	<.001	<.001
	conjunctivitis	<.001	0.06 (0.04)	13 (11)	<.001		<.001	<.001	<.001	<.001		<.001	<.001	<.001
	ear infection	<.001	0.07 (0.05)	4 (18.6)	<.001		.05	.001	<.001	<.001		.05	<.001	<.001
	fifth disease	<.001	0.14 (0.06)	11 (2.5)	<.001		.03	.02	<.001	<.001		.03	.02	<.001
	german measles	.38	—	—	.71		.91	.81	.21	.97		.90	.63	.14
	gonorrhea	.48	—	—	<.001		.01	<.001	.02	<.001		.01	<.001	.01
	HIV	.05	0.02 (0.03)	13 (14.8)	<.001		<.001	<.001	<.001	<.001		<.001	<.001	<.001
	hpv	.62	—	—	<.001		.15	.09	<.001	<.001		.14	.07	<.001
	impetigo	.03	0.07 (0.06)	35 (11)	<.001		<.001	<.001	<.001	<.001		<.001	<.001	<.001
	lice	<.001	0.06 (0.05)	35 (9.8)	<.001		<.001	<.001	<.001	<.001		<.001	<.001	<.001
	measles	.01	0.23 (0.07)	12 (3.6)	.10		.01	.06	<.001	.08		.01	.05	<.001
	meningitis	.62	—	—	<.001		.008	<.001	<.001	<.001		.008	<.001	<.001
	mononucleosis	.25	—	—	<.001		.77	.55	<.001	<.001		.75	.44	<.001
	mumps	.62	—	—	.01		.004	.002	<.001	.01		.004	.002	<.001
	pertussis	.64	—	—	<.001		.84	.36	.003	<.001		>.99	.28	.002
	pink eye	<.001	0.09 (0.06)	9 (17.4)	<.001		<.001	<.001	<.001	<.001		<.001	<.001	<.001
	ringworm	<.001	0.03 (0.04)	27 (10.6)	.04		<.001	<.001	<.001	.03		<.001	<.001	<.001
	rubella	.01	0.05 (0.05)	12 (14.5)	.15		.69	.79	.01	.12		.65	.62	.008
	salmonella	.62	—	—	.25		.45	.43	<.001	.20		>.99	.35	<.001
	scabies	.11	—	—	<.001		.02	<.001	<.001	<.001		.02	<.001	<.001
	shingles treatment	.19	—	—	.15		.47	.33	.93	>.99		>.99	>.99	.62
	std	.02	0.01 (0.03)	20 (15.9)	<.001		<.001	.005	<.001	<.001		<.001	.004	<.001
	stomach flu	<.001	0.33 (0.06)	3 (1.2)	<.001		<.001	<.001	<.001	<.001		<.001	<.001	<.001
	strep throat	<.001	0.12 (0.07)	5 (19.9)	<.001		<.001	<.001	<.001	<.001		<.001	<.001	<.001
	syphilis	.88	—	—	.22		.81	.36	.93	.17		.78	.29	.70
	tuberculosis	.05	—	—	.53		.04	.74	.02	.40		>.99	.60	.02
**Noncommunicable, control conditions (non-COVID)**														
	arthritis	.26	—	—	<.001		.33	.65	.07	<.001		>.99	>.99	.05
	broken bone	.23	—	—	<.001		.03	.53	.004	<.001		.03	.43	.003
	burning eyes	>.99	—	—	<.001		.002	.04	<.001	>.99		>.99	>.99	>.99
	cancer	.26	—	—	<.001		<.001	.007	<.001	<.001		<.001	.006	<.001
	cataracts	.97	—	—	<.001		.33	.50	.12	<.001		.30	.41	.08
	claritin	<.001	0.16 (0.06)	18 (3.2)	.50		.63	.59	.002	.39		>.99	.46	.002
	corneal ulcer	.26	—	—	.10		.04	.74	.86	.08		.03	.60	.56
	diabetes	.01	0.02 (0.05)	6 (18.9)	<.001		.84	.20	.93	<.001		>.99	.16	.70
	diabetic retinopathy	.88	—	—	.15		.70	.43	.40	.12		>.99	>.99	.26
	dry eyes	.13	—	—	.02		.93	.04	<.001	.02		>.99	>.99	>.99
	eczema	<.001	0.04 (0.05)	9 (17.2)	.30		.19	.04	.003	.24		.18	.03	.002
	glaucoma	.41	—	—	<.001		.42	.36	.03	<.001		.38	.29	.02
	heart attack	.01	0.04 (0.06)	12 (10)	.15		.41	.34	.03	.12		.37	.28	.02
	high blood pressure	<.001	0.05 (0.04)	5 (13.2)	<.001		.91	.66	.29	<001		.89	>.99	.20
	itchy eyes	<.001	0.1 (0.05)	18 (5.3)	<.001		.003	.10	.22	>.99		>.99	>.99	>.99
	macular degeneration	.78	—	—	<.001		.42	.36	.08	<.001		>.99	>.99	.05
	memory loss	.92	—	—	<.001		.81	.79	.93	<.001		>.99	.62	.71
	pollen	<.001	0.29 (0.07)	17 (1.7)	<.001		<.001	<.001	.28	>.99		>.99	>.99	>.99
	pregnant	.05	—	—	.38		.43	.79	.80	.30		.38	.62	.53
	red eyes	.19	—	—	.004		.72	.86	.59	>.99		>.99	.66	.39
	sore eyes	.83	—	—	.51		.22	.04	.12	.39		>.99	>.99	>.99
	stroke symptoms	<.001	0.09 (0.04)	26 (8)	.25		.91	.36	.06	>.99		>.99	>.99	>.99
	toothache	.62	—	—	.06		.41	.13	.34	>.99		.37	.11	.23

^a^Indicates if the search for the years 2011-2019 shows a significant (*P*<.05) seasonal trend.

^b^Two-sided *P* values regarding any change in search from prior years for each season.

^c^One-sided *P* values regarding a decrease in search from prior years for each season.

^d^AtM: amplitude to mean ratio, indicating relative seasonal strength.

^e^Indicates peak high season.

^f^Not applicable; AtM and circular mean values are provided only for search terms where statistical evidence of that term being seasonal was found.

**Table 3:** Comparison of the differences and reductions in search postpandemic (*P* values in Table 2), for communicable vs noncommunicable condition search terms groups, by season.

Season	Difference from prior years^a^		Search lower than prior years^b^	
	Wilcoxon rank-sum test	Fisher exact test	Wilcoxon rank-sum test	Fisher exact test
Spring	.99	.83	.04	.09
Summer	.05	.01	<.001	<.001
Autumn	.02	.01	<.001	<.001
Winter	<.001	.003	<.001	<.001

^a^*P* values when testing if significant changes in search after the start of the pandemic differed for the communicable and noncommunicable classifications of non-COVID-19 term groups.

^b^*P* values when testing if significant reductions in search after the start of the pandemic differed for the communicable and noncommunicable classifications of non-COVID-19 term groups.

### Seasonal Characteristics and Their Relationship to Reductions in 2020-2021

Although we found searches for a number of terms from all 3 classifications that appeared to be seasonal, it appeared that seasonal terms were more likely to have a reduced search frequency in 2020-2021 seasons (see panels in Figure 2, including the black text on the lower left of all panels and Table 2 “Seasonality” *P* values). We hypothesized that seasonal conditions might be reduced by social distancing measures during the pandemic more than for those that are less seasonal. To test this hypothesis, for each season of each non-COVID-19 term, we compared the *P* values for search reduction against the seasonality *P* values for that term using Theil-Sen regression. For spring, we found no significant correlation between a term having reductions in search in 2020-2021 and with the seasonality of a term (*P*=.95). However, for summer, autumn, and winter, we found a significant correlation between a term having reductions in search in 2020-2021 with the seasonality of that term (Theil-Sen: summer *P*<.001, autumn *P*<.001, winter *P*<.001).

## Discussion

### Principal Results

#### Decreased Searches for Communicable and Seasonal Disease Search Terms During 2020-2021

Overall, in our masked analysis, searches for many of the 29 non-COVID-19 communicable terms (including those related to conjunctivitis) were significantly decreased during the first 4 seasons of the 2020-2021 pandemic compared with the prior 9 years. For example, 18 of the terms (*“chicken pox,” “chlamydia,” “cold medicine,” “conjunctivitis,” “ear infection,” “fifth disease,” “gonorrhea,” “HIV,” “impetigo,” “lice,” “meningitis,” “mumps,” “pink eye,” “ringworm,” “scabies,” “std,” “stomach flu,” “strep throat”*) showed reductions for all 4 seasons of the pandemic (see Table 2). For 3 consecutive seasons in 2020-2021 (summer, autumn, winter), the levels of search were much more significantly reduced in 2020-2021 for the non-COVID-19 communicable terms group than for the noncommunicable terms group. The conjunctivitis-related findings of sustained reduction in search continue to lend support to our hypothesis described in our prior study from the start of the pandemic, based on reduced searches for conjunctivitis terms, that social distancing from the pandemic may lead to reductions in infectious conjunctivitis [5]. Recently, Lavista Ferres et al [56] provided support of this hypothesis, demonstrating that a 37% decrease in emergency department encounters for infectious conjunctivitis was associated with implementation of social distancing, reduced smartphone mobility, and reduced online search. Our results also support a broader hypothesis that non-COVID-19 communicable disease in general may be reduced in comparison to control noncommunicable conditions due to implementation of social distancing. In a separate assessment independent of our search term classifications, we also found a significant correlation between reductions in search for a term in 2020-2021 and seasonality of search for that term. This is not surprising, as it appears that many terms of communicable conditions were seasonal and with apparent higher seasonality overall compared to noncommunicable conditions.

#### Increase of Searches With Non-COVID-19 Ocular Terms During the Pandemic

Of the terms we had initially classified as not clearly COVID-19 pandemic–related and as noncommunicable, the only terms that showed significant increases in 2020-2021 for one or more seasons included “*pollen*” and several ocular terms (*“burning eyes,” “dry eyes,” “itchy eyes,” “red eyes,” “sore eyes”*). Despite this, no other control ocular conditions (*“cataracts,” “corneal ulcer,” “diabetic retinopathy,” “glaucoma,” “macular degeneration”*) were significantly increased. This suggests that unlike communicable ocular conditions, which had a lower search during the pandemic (conjunctivitis), or noncommunicable chronic ocular conditions (without a sustained change in search), these other ocular conditions may have indeed increased during the pandemic. This appears most likely for “*burning eyes*” as well as “*dry eyes*” and “*itchy eyes.*” These findings lend support to some clinical studies (although not all of them draw the same conclusions) suggesting that some of these elevated ocular symptoms may be linked to COVID-19 or to other impacts of the pandemic, such as mask-wearing and increased screen time [5,28-36,41]. For example Nasiri et al [28] found common ocular manifestations in patients with COVID-19, including dry eye, redness, tearing, itching, eye pain, and discharge, and Moshirfar et al [33] reported that facemask wearing may cause ocular irritation and dryness in regular mask wearers.

#### Sustained Decrease in Searching Non-COVID-19 Noncommunicable Terms During the Pandemic

A few noncommunicable terms had sustained search reductions in 2020-2021. Search for “*cancer*” was reduced for all 4 seasons compared to prior years. Chen et al [18] reported declines in colorectal, prostate, and breast cancer screening rates with the start of the COVID-19 pandemic through mid-summer. It is possible that fewer positive results from screening of healthy adults could potentially have led to fewer people searching for “*cancer.*” For some terms in our communicable condition group representing conditions covered by routine annual clinical screening (such as “*std*”), the decreased search may therefore also reflect less screening services or test results rather than a reduced prevalence. Johnson et al [20] reported large declines in STD testing and in STD programmatic operations during the first 6 months of the pandemic. Our observed sustained reduction in search for bone fracture (“*broken bone*”) reflects what has also been observed clinically during the pandemic. For example, one systematic review reported a 43% decline in the number of fractures presenting to hospitals during the pandemic compared to prepandemic levels that they attributed to less driving, sports, and other outdoor activities during the pandemic [19].

#### Impact of the Pandemic on Searching for COVID-19 and Influenza Terms

Unlike the non-COVID-19 groups, in several seasons of 2020-2021, most search terms in the group we had classified as related to COVID-19 had significant search increases. An exception was that earlier increases in search for influenza-related terms reversed in winter to become significant decreases. This could indicate that early on in the pandemic, COVID-19 symptoms may have been misconstrued as being related to flu [57] or that searches to distinguish COVID-19 from flu were common. By winter 2020, these reasons may have waned, while, in parallel, an actual drop in flu cases (and therefore less flu searches) may have occurred due to social distancing during the peak flu season. This has been suggested from clinical data as well. For example, a systematic review performed by Fricke et al [57] showed that defined influenza cases and influenza positivity rate were lower during the pandemic than in former seasons.

### Limitations

As with many infodemiology studies, it is possible that multiple other causes can affect search trends besides the occurrence rate of a disease. We may expect this for some terms such as those related to conditions reported in the news during the pandemic. However, the fact that our general finding of more reduced search for communicable than noncommunicable terms suggests that this is not the case globally. Furthermore, a search reduction due to news stories is much less likely than an increase. Reduced search for a term related to news about that term also would not likely be sustained for several seasons. Many of our health terms exhibited a general overall search reduction in spring 2020 (other than those potentially related to COVID-19). Those in the noncommunicable group tended to return to normal levels by summer 2020. This may indicate that seeking medical care for these other conditions was reduced due to public concern of going to clinics as well as closed clinics. Some terms had no significant changes noted during the pandemic compared to prior years. This could reflect unchanged clinical conditions. Alternatively, the search volume for some terms may be too low overall, preventing determining statistical significance using our methods.

For a small number of terms, although searches in 2020-2021 visually appeared to be lower than in prior years, they were not shown to be significantly different than those in prior years. This could be due to our analysis accounting for secular trends already moving in that direction (eg, see the red line in the “*pregnant*” panel of Figure 2). Indeed, other epidemiological studies have seen a decrease during the pandemic that was also partially obscured by a prior secular trend [58,59].

### Comparison With Prior Work and Significance

This study lends support to our prior study hypotheses, and confirms theoretical public health and epidemiological assumptions about the value of social distancing to reduce the impact of conjunctivitis epidemics [5,56,60]. It also builds upon and complements a growing body of evidence from clinical and other epidemiologic studies suggesting that social distancing and public health interventions such as school closures during the pandemic can potentially reduce the prevalence of numerous other communicable diseases, including pediatric respiratory tract infection, non-COVID-19 acute pediatric infections, varicella, measles, rubella, head lice, influenza, and STDs, as well as other condition [12-17,19-21,56].

Use of online information-seeking behavior data to infer changes in disease can be simultaneously applied to entire countries, states, and smaller regions, and to numerous conditions, potentially worldwide. Being able to leverage such low-cost early monitoring can help detect or predict clinical or epidemiological status or outcomes early on during an event to potentially allow for improved modeling and planning by public health programs. Such approaches could be considered to complement findings from clinical studies and to reveal findings prior to availability of clinical data, such as what occurred during the COVID-19 pandemic. Indeed, an early study of reduced disease term search data, at a time when clinical data were unavailable, suggested that one cause was due to a potential decline in clinical cases, which, many months later, was confirmed from clinical data [5,56].

### Conclusions

Compared to studies based on more costly and less publicly available individual-level clinical data, we demonstrate the use of online search data to study the impacts of interventions such as social distancing at very low cost. Results from the study of online search data lend support to emerging clinical evidence implicating social distancing and the COVID-19 pandemic in the reduction of communicable disease and in the impact on ocular conditions.

## Abbreviations

API: application programming interface
AtM: amplitude-to-mean ratio
NIH-NEI: National Institutes of Health National Eye Institute
STD: sexually transmitted disease
